# 

**Published:** 1884-12

**Authors:** 


### End of the Volume.

With this Number, the Thirty-eighth Volume of the REGISTER is completed. Of what it contains we need not now speak, for those who have it know, and those who have not, can hardly know what they have missed. The REGISTER does not claim to be the embodiment of all that is good and valuable in the profession, but it has endeavored to make a record of much that has transpired during the last year; making record, as far as it can, of such things as may indicate the advance of the profession. What it may be in the future, we can not say. Certainly, so far as the Editors are concerned, the aim is to make it equal, if not better than in the past. We shall hope to have the co-operation of the profession in the accomplishment of this purpose. This co-operation will make the journal better in some respects, so we trust that the profession will consider that there is a standing invitation to present their thoughts through this medium. Our established periodical literature should have the co-operation of the profession. The labor of those who have it immediately in charge will then be heavy enough; but if the whole burden is to be borne by the few, it may be more than can be well sustained. Give us, then, your best thoughts, accurate descriptions, and best methods and processes, and you will thus minister to the advance of the profession.

### Wanted.

Three or four copies of the Transactions of the American Dental Association, for the year 1870. Also one copy each of the following years: 1859-60-62-63, and 1876. Any one having these copies, and willing to part with them, will please send them to the Editor of the DENTAL REGISTER, for which ample

### OHIO COLLEGE OF DENTAL SURGERY,

No. 29 College St., Cincinnati, O.

### THIRTY-EIGHTH ANNUAL SESSION, 1883-4.

### FACULTY.

CHAS. KEARNS, M. D.,

*Anatomy and Oral Surgery.*

FRANK BELL, D. D. S.,
*Mechanical Dentistry and Metallurgy.*

J. S. CASSIDY, M. D., D. D. S.,
*Chemistry and Materia Medica.*

C. M. WRIGHT, D. D. S.,

*Physiology and General Pathology.*

H. A. SMITH, D. D. S., Dean of the Faculty,
*Operative Dentistry and Dental Pathology.*

G. W. KEELY, D. D. S.,
*Lecturer on Causes and Management of Irregularities of the Teeth.*

### DEMONSTRATORS.

A. E. McCONKEY, D. D. S., *Demonstrator of Operative Dentistry.*

WM. KNIGHT, M. D., *Demonstrator of Anatomy.*

G. S. JUNKERMAN, D. D. S., *Demonstrator of Analytical Chemistry.*

GRANT MOLLYNEAUX, D. D. S., *Demonstrator of Mechanical Dentistry.*

This Institution is a DENTAL COLLEGE IN THE STRICTEST SENSE. The property is owned by an Association of Dentists, numbering one hundred. The Faculty is composed of Practitioners of Dentistry, whose purpose it is to give a thorough course of instruction in the theory and practice of Dentistry.

The College Building was designed, erected, and is exclusively used for a Dental School. The reception, lecture, clinical and dissecting rooms, and mechanical laboratory are large, well lighted, and admirably arranged for the purposes of dental education. Located in the center of one of our most populous cities, where there is no other Dental School, the supply of patients is abundant, and at times in excess of the requirements for clinical practice. The College Infirmary will be open every afternoon (except Sundays), during the session, thus affording the students ample opportunities to engage in ACTUAL PRACTICE under the direction of the Professors and Demonstrators.

### GRADUATION.

The candidate must be twenty-one years of age. He must have attended two courses of lectures in a Dental or Medical College, the last of which must be in this Institution. A reputable dentist in actual practice five years, may be examined for the degree of D. D. S., after attending one full course of lectures.

### FEES.

| | |
|---|---|
| Matriculation fee (but once) | $ 5 00 |
| Professors' tickets for one session (whether Junior or Senior) | 75 00 |
| Professors' tickets for second year | 50 00 |
| Dissecting ticket, including material | 10 00 |
| Demonstrator of Chemical Analysis | 10 00 |
| Diploma Fee | 25 00 |

The session begins the 10th of October, and continues until the first of March. Board can be had for from $4.00 to $6.00 per week.

For further information, address

**H. A. SMITH, Dean,**
**286 Race Street, CINCINNATI.**

### THE DENTAL COLLEGE

OF THE

### University of Michigan.

The Ninth Annual Session of this Institution will commence on the 1st of October and close on the last Wednesday of March, thus making a course of six months. The regular course of instruction commences at once, and will proceed through the term, with the customary vacation.

The students in this department will receive instruction in Anatomy, Physiology, Pathology, Chemistry, Materia Medica, Therapeutics, and Surgery, from the Professors of their respective branches in the *Department of Medicine and Surgery* of the University, when lectures commence, and continue the same as with the Dental College. There will also, in addition, be a special course upon each of these branches. The courses will each embrace from ten to fifteen lectures.

### FACULTY OF THE DENTAL DEPARTMENT.

**J. B. ANGELL, LL.D.** - - - - - - - - - - - - **President.**
**J. TAFT, M.D., D.D.S.** **Principles and Practice of Operative Dentistry.**
**CORYDON L. FORD, M.D., D.D.S.** - - - **Anatomy and Physiology.**
**J. A. WATLING, D.D.S.** - - - **Clinical and Mechanical Dentistry.**
**W. H. DORRANCE, D.D.S.** - - - - - - **Prosthetic Dentistry.**
**U. D. BILLMEYER, D.D.S.** - - **Demonstrator of Clinical Dentistry.**
**C. S. CASE, D.D.S.** - - - - **Demonstrator of Prosthetic Dentistry.**

Special instruction will be given in Dental Pathology, Oral Surgery, Dental Therapeutics, and Diseases of Women and Children, with reference to the teeth.

Students should be promptly present at the College on Friday, September 28th, at 10 o'clock, A.M., to make the preliminary arrangements for entering upon regular work. Seats in the lecture room are assigned by selection to students in the order of registration on the Steward's Books; and each student is expected to occupy during the session such seat as he may select. Students, on arriving in Ann Arbor, should call at the Steward's office.

### CONDITIONS OF GRADUATION.

The candidate must be twenty-one years of age. *He must furnish satisfactory evidence of good moral character.*

He must devote three years to the study of his profession. He must attend two full courses of lectures in the Dental College, or one course in some college having an equal standard of requirements, and the last one here, and we recommend that he attend three courses regularly.

He must sustain an examination satisfactory to the Faculty in all the branches taught.

A graduate of the Medical College may enter the Senior Class, and, if found qualified, may graduate after two years have been devoted to the study of dentistry.

### FEES AND EXPENSES.

The fees, which must be paid in advance, are as follows:

Residents of Michigan.—Matriculation fee, $10.00; annuel dues, $20.00.

Non-Residents.—Matriculation, $25.00; annuel dues, $25.00.

Graduation Fee.—For all alike, $10.00. The admission fee is paid but once, and entitles the student to the privileges of permanent membership in any department of the University. The annual dues are paid the first year and every year thereafter while at the University.

For further particulars, address the Dean of the Dental College, Ann Arbor, Michigan.

**J. TAFT, Dean.**

Aug-83-6m.

### CONTENTS.

COMMUNICATIONS.

### THE DENTAL REGISTER,

A MONTHLY MAGAZINE DEVOTED TO THE INTERESTS OF THE

**DENTAL PROFESSION.**

*Terms Reduced to* $2.00 *Per Year. Single Copies,* 25 *Cents.*

**EDITED BY PROF. J. TAFT, D.D.S.**

AND

**Published by SPENCER & CROCKER, Ohio Dental and Surgical Depot.**

The volume commences in January and closes in December.

The REGISTER being issued monthly is always fresh, therefore Indispensable to the practitioner who desires to keep posted up with the new inventions and improvements which are daily developed. Neither expense nor effort will be spared to give value and variety to its contents Articles will be illustrated with well executed engravings whenever they will add to the interest or assist to explanation.

Its extensive circulation and frequency of issue make it one of the BEST DENTAL ADVERTISING MEDIUMS in the United States.

All subscriptions must begin with January or July.

Local or special notices, five lines or less, will be inserted for $3 each insertion; over five lines, 50 cts. per line.

All advertising bills payable in advance, or at furthest, after the issue of the first number containing the advertisement. All transient advertisements to be paid for in advance.

☞CONTRIBUTIONS SOLICITED.

Exclusively Editorial Communications should be addressed to the Editor.

The latest number of the REGISTER may be ordered with or without other goods at any time, and all letters should be addressed to

**SPENCER & CROCKER, Ohio Dental & Surgical Depot, Cincinnati, O.**

